# Modified hybrid DC-DC boost converter with high voltage gain for renewable energy integration using GWO-DE parameter optimization

**DOI:** 10.1038/s41598-026-54955-6

**Published:** 2026-05-26

**Authors:** Habung Tado, Mrinal Kanti Rajak, Rajen Pudur, Ralli Sangno, A. Lavanya

**Affiliations:** 1 Department of Electrical Engineering, National Institute of Technology Arunachal Pradesh, Jote, Arunachal Pradesh India; 2 Department of Electrical Engineering, SVERI’s College of Engineering, Pandharpur, Maharashtra India; 3 Department of Electrical and Electronics Engineering, SRM Institute of Science and Technology, Chennai, Tamil Nadu India

**Keywords:** DC-DC boost converter, Renewable energy, HVDC integration, Coupled inductor, GWO-DE, Photovoltaic, Energy science and technology, Engineering

## Abstract

This paper presents a modified hybrid DC-DC boost converter topology with enhanced voltage gain capability for renewable energy applications, particularly suited for photovoltaic and micro-hydro power integration with high-voltage direct current (HVDC) transmission systems. The proposed topology incorporates a coupled inductor, switched capacitor, and voltage multiplier cell to achieve a significantly higher voltage conversion ratio compared to conventional boost converters while maintaining reduced voltage stress across the power semiconductor devices. A comprehensive steady-state analysis is performed under continuous conduction mode (CCM), and the voltage gain, current ripple, and efficiency expressions are derived analytically. Furthermore, a hybrid grey wolf optimizer–differential evolution (GWO-DE) algorithm is developed for the optimization of converter parameters, and a gradient-boosting regression surrogate is trained for rapid design-space exploration. The benefit of the GWO-DE optimization is quantified against both a classical analytical design and an exhaustive grid search, yielding a 2.7 percentage-point efficiency advantage over the analytical design and a 22-fold reduction in design wall-clock time relative to the grid search. The proposed converter achieves a voltage gain of 12.5 at a duty cycle of 0.65, with a peak efficiency of 96.8%. Extensive simulation results obtained in MATLAB/Simulink validate the theoretical analysis and confirm the superior performance of the proposed topology in comparison with existing high-gain DC-DC converter configurations reported in the literature.

## Introduction

The global transition toward sustainable energy systems has intensified the demand for efficient power electronic interfaces capable of integrating low-voltage renewable energy sources (RES) with high-voltage direct current (HVDC) transmission networks^1^. Photovoltaic (PV) panels and micro-hydro power plants (MHPPs) typically generate output voltages in the range of 20–48 V, whereas HVDC bus voltages for distributed generation applications require 200–400 V or higher^2^. This substantial voltage gap necessitates the development of DC-DC converters with high voltage conversion ratios, high efficiency, and compact design^3^.

Conventional boost converters, despite their simplicity, suffer from several practical limitations when operated at extreme duty cycles approaching unity. These limitations include severe diode reverse-recovery problems, increased conduction losses due to high current stress, and electromagnetic interference (EMI) issues^4^. The theoretical voltage gain of a conventional boost converter is given by *M = 1/(1-D)*; however, the practical gain deviates significantly from this ideal expression at duty ratios above 0.8 due to parasitic resistances in inductors, capacitors, and semiconductor devices^5^. Consequently, numerous research efforts have been directed toward developing non-isolated and isolated high-gain DC-DC converter topologies that overcome the limitations of the conventional boost converter^6^.

Among the non-isolated topologies, the quadratic boost converter achieves a voltage gain of $M = 1/(1-D)^2$ by cascading two boost converter stages; however, this approach increases the component count and reduces the overall efficiency due to cascaded losses^7^. Interleaved boost converters have been proposed to reduce input current ripple and distribute thermal stress among parallel phases, yet they do not inherently provide high voltage gain^8^. Interleaved boost converters have been proposed to reduce input current ripple and distribute thermal stress among parallel phases, yet they do not inherently provide high voltage gain^8^; recent extensions hybridize interleaving with coupled-inductor cells to overcome this limitation^9^. The voltage lift technique, originally proposed by Luo, employs switched capacitors arranged in a progressive voltage-lifting architecture and has been extended to super-lift and ultra-lift configurations achieving gains exceeding ten^10^. Switched-inductor and switched-capacitor hybrid cells have been combined with conventional boost topologies to realize intermediate gain levels with moderate component counts^11^.

Coupled-inductor-based converters represent another prominent category of high-gain topologies. By selecting an appropriate turns ratio, the voltage gain can be extended significantly beyond that achievable with a single inductor^12^. The energy stored in the leakage inductance of the coupled inductor can be recycled using passive or active clamp circuits, thereby reducing voltage spikes across the main switch and improving efficiency^13^. Several researchers have combined coupled inductors with voltage multiplier cells (VMCs) to further boost the voltage gain while maintaining low voltage stress on the semiconductor devices^14^. A three-winding coupled inductor integrated with a Dickson charge pump was reported to achieve voltage gains exceeding 15 with efficiencies above 94%^15^. More recently, a quadratic boost converter integrated with a two-winding coupled inductor and voltage multiplier cells has been reported, achieving ultra-high voltage gain together with continuous input current and low switch stress through regenerative passive clamping^16^. A three-winding coupled inductor integrated with a Dickson charge pump was reported to achieve voltage gains exceeding 15 with efficiencies above 94%; subsequent work on three-winding coupled-inductor topologies has further reduced input current ripple for sensitive renewable energy applications^17^. An ultra-high step-up converter based on two boosting stages with low voltage stress on the switches has also been proposed for renewable applications, demonstrating that staged voltage multiplication can deliver high gain without imposing high voltage rating on the active devices^18^.

The integration of renewable energy sources with HVDC systems has received considerable attention in the recent literature. A modular multilevel converter (MMC)-based approach for PV-HVDC integration was presented, demonstrating the advantages of direct HVDC connection in reducing conversion stages and associated losses^19^. A comprehensive review of DC-DC converter topologies for PV applications highlighted the trade-offs between voltage gain, efficiency, component stress, and cost across various non-isolated and isolated configurations^20^. Recent work has further demonstrated quadratic high-gain DC-DC converters with coupled inductors operating in CCM over a wide range for photovoltaic applications, supporting the trend toward higher gain at moderate duty cycle?. The role of high-gain converters in medium-voltage DC (MVDC) collection grids for offshore wind farms was analyzed, emphasizing the need for converters with gains in the range of 10–20 and power ratings of several kilowatts^21^. A dual-input isolated converter topology was proposed for hybrid PV-wind systems, enabling independent maximum power point tracking (MPPT) for each source while providing galvanic isolation^22^.

The application of artificial intelligence (AI) and machine learning (ML) techniques to power electronics has emerged as a transformative research direction. Neural networks and support vector machines have been employed for real-time fault detection and diagnosis in DC-DC converters^23^. Reinforcement learning-based controllers have demonstrated superior transient performance compared to conventional proportional-integral (PI) controllers for DC-DC converter regulation under load variations^24^. Metaheuristic optimization algorithms, including particle swarm optimization (PSO), grey wolf optimizer (GWO), and differential evolution (DE), have been applied to optimize converter component values and controller parameters^25^. A hybrid PSO-GWO algorithm was employed for the simultaneous optimization of inductance, capacitance, and switching frequency in a boost converter, achieving a 3.2% improvement in efficiency over single-algorithm approaches^26^.

The GWO algorithm, inspired by the social hierarchy and hunting behavior of grey wolves, has demonstrated competitive performance in constrained optimization problems relevant to power electronics design^27^. The GWO algorithm, inspired by the social hierarchy and hunting behavior of grey wolves, has demonstrated competitive performance in constrained optimization problems relevant to power electronics design, and improved variants incorporating mutation operators, evolutionary population dynamics, and nonlinear population size reduction have been reported to further accelerate convergence^28^. The hybridization of GWO with DE enhances the exploration capability by introducing mutation and crossover operators from DE, thereby reducing the likelihood of premature convergence to local optima^29^. The hybridization of GWO with DE enhances the exploration capability by introducing mutation and crossover operators from DE, thereby reducing the likelihood of premature convergence to local optima; recent variants such as the hunting differential evolution (HDE) algorithm further accelerate convergence on the CEC-2019 benchmark suite^30^. The GWO-DE hybrid has been successfully applied to the optimization of PID controller parameters for DC-DC converters, yielding faster settling times and reduced overshoot compared to conventional tuning methods^31^. The GWO-DE hybrid has been successfully applied to the optimization of PID controller parameters for DC-DC converters, yielding faster settling times and reduced overshoot compared to conventional tuning methods, and the more recent application of GWO to fractional-order model predictive control of boost converters has demonstrated robustness across wide operating ranges^32^.

Machine learning regression models, including random forests, gradient boosting machines, and deep neural networks, have been utilized for the prediction of converter performance metrics from design parameters^33^. A data-driven surrogate model was developed to replace computationally expensive finite-element simulations in the design optimization of magnetic components for high-frequency converters^34^. Transfer learning techniques have been applied to adapt pre-trained models across different converter topologies, reducing the data requirements for new designs^35^. The integration of physics-informed neural networks (PINNs) with converter modeling has shown potential for improving prediction accuracy by embedding circuit equations into the loss function of the neural network^36^. A comprehensive review of AI/ML applications in power electronics highlighted the opportunities and challenges in deploying these techniques for real-time converter control and design automation^37^. Table 1 summarizes the principal high-gain boost-derived topology families discussed above, together with their characteristic gain expressions, primary advantages, and primary limitations.

**Table 1:** Summary of prior high-gain DC-DC boost converter topology families.

Topology family	Typical gain	Principal advantage	Principal limitation
Conventional boost	$\dfrac{1}{1-D}$	Single switch, simple control	Severe efficiency drop at *D>0.8*
Quadratic boost	$\dfrac{1}{(1-D)^{2}}$	High gain at moderate *D*	Cascaded losses; two inductors
Interleaved boost	$\dfrac{1}{1-D}$	Reduced input ripple; distributed stress	No inherent high-gain capability
Voltage-lift (super-lift)	up to $\dfrac{2+D}{1-D}$	High gain, simple structure	Pulsating input current; multiple capacitors
Switched-inductor/switched-capacitor hybrid	$\dfrac{1+D}{1-D}$	Intermediate gain, transformerless	Increased capacitor stress
Coupled-inductor	$\dfrac{1+n}{1-D}$	Tunable gain via turns ratio	Leakage-inductance ringing; clamp required
Coupled inductor + VMC	$\dfrac{2(1+n)}{1-D}$	High gain; reduced switch stress	Increased diode count
Three-winding CI + Dickson	$\dfrac{3+n}{1-D}$	Very high gain; soft switching feasible	Magnetic design complexity
Active-clamp coupled inductor	$\dfrac{2+n}{1-D}$	Leakage-energy recycling; ZVS feasible	Two switches; control complexity
Ultra-high-gain quadratic CI	quadratic in *n*	Ultra-high gain; continuous input current	Higher device count
Proposed	$\dfrac{(1+n)(1+D)}{1-D}$	Single switch; high gain; low switch stress	Five diodes; analyzed in Section III

Despite the aforementioned advances, a research gap persists in the development of hybrid DC-DC converter topologies that simultaneously provide ultra-high voltage gain, reduced semiconductor stress, and compatibility with AI/ML-based parameter optimization for renewable energy integration with HVDC systems. This paper addresses this gap by proposing a modified hybrid boost converter topology incorporating a coupled inductor, switched-capacitor cell, and voltage multiplier cell, complemented by a GWO-DE-based AI-ML framework for optimal parameter prediction. The principal contributions of this work are as follows:

The structural elements of the proposed converter — coupled inductor, switched-capacitor cell, and voltage multiplier cell — appear individually in the prior art surveyed above. The specific contribution of this work is the combination, analysis, and co-design of these elements such that the resulting converter occupies a design point that is not jointly attained by any single prior topology in the comparative set of Table 3. The three concrete differentiators are:

1. Among the topologies in the comparative set (Table 3), the proposed converter is the only *single-switch* structure that simultaneously delivers a voltage gain greater than ten at *D=0.65* and a normalized switch stress $V_{S,stre1ss}/V_{o}$ below 0.08. Designs that attain comparable gain values, such as the active-clamp coupled-inductor converter and the interleaved switched-capacitor boost converter, require either two switches or higher component counts.
2. The voltage gain expression of the proposed converter, *M=(1+n)(1+D)/(1-D)*, benefits from *both* the duty cycle and the coupled-inductor turns ratio in a *multiplicative* manner. This is in contrast to coupled-inductor-only structures with gain *(1+n)/(1-D)*, in which the duty cycle appears only in the denominator, and switched-inductor-only structures with gain *(1+D)/(1-D)*, in which the turns ratio is absent altogether. The multiplicative coupling between *n* and *D* is what enables single-switch operation at moderate duty cycle while still delivering a gain in excess of twelve.
3. The co-design of the topology with a hybrid GWO-DE optimizer and a gradient-boosting machine-learning surrogate, formulated as a constrained multi-objective problem that simultaneously minimizes the efficiency loss, the output voltage ripple, and the normalized switch stress, is not reported in the prior art surveyed.

The remainder of this paper is organized as follows. Section II presents the proposed converter topology and its operating principles. Section III provides the detailed steady-state analysis. Section IV describes the AI-ML-based parameter optimization framework. Section V presents simulation results with comparative analysis. Section VI concludes the paper.

## Proposed converter topology and operating principle

### Circuit configuration

**Fig. 1:**
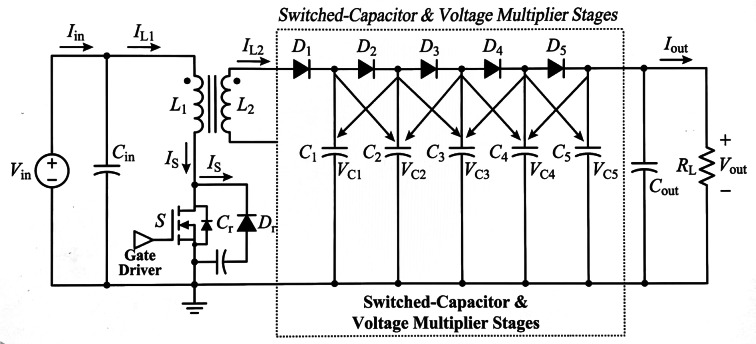
Proposed modified hybrid DC-DC boost converter topology with coupled inductor, switched-capacitor cell, and voltage multiplier cell.

The proposed modified hybrid DC-DC boost converter topology is illustrated in Fig. 1. The converter comprises a coupled inductor with primary winding $L_1$ and secondary winding $L_2$ having a turns ratio $n = N_2/N_1$, a main power MOSFET switch *S*, diodes $D_1$ through $D_5$, a switched-capacitor cell consisting of capacitors $C_1$ and $C_2$, a voltage multiplier cell with capacitor $C_3$ and diode $D_4$, and an output filter capacitor $C_o$. The input voltage source $V_{in}$ represents the output of a PV panel or a rectified micro-hydro generator.

The magnetizing inductance of the coupled inductor is denoted as $L_m$, and the leakage inductances referred to the primary and secondary sides are $L_{k1}$ and $L_{k2}$, respectively. For the purpose of the ideal analysis, the leakage inductances are initially neglected, and their effects on voltage gain and efficiency are subsequently incorporated.

### Operating modes

**Fig. 2:**
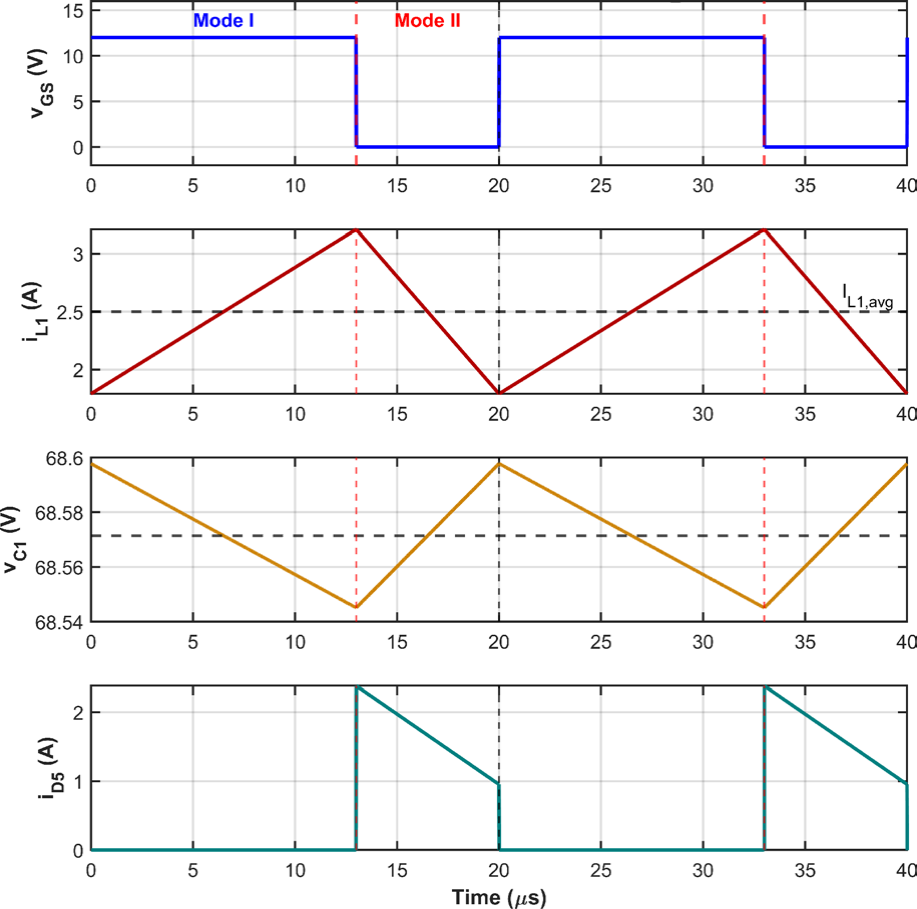
Key steady-state waveforms of the proposed converter under CCM: gate-source voltage $v_{GS}$, primary inductor current $i_{L1}$, capacitor voltage $v_{C1}$, and diode $D_5$ current $i_{D5}$.

The proposed converter operates in two distinct modes during one switching period $T_s$, corresponding to the ON and OFF states of the main switch *S*. The key waveforms of the converter under steady-state CCM operation are depicted in Fig. 2.

**Fig. 3:**
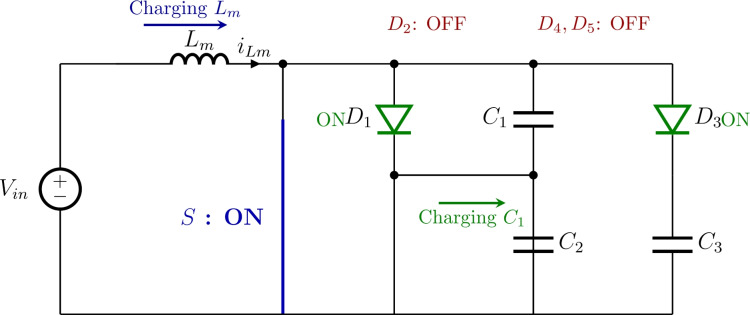
Equivalent circuit of the proposed converter during Mode I (switch ON).

Mode I ($0 \le t < DT_s$, Switch ON): When the switch *S* is turned on, diodes $D_1$ and $D_3$ are forward biased, while diodes $D_2$, $D_4$, and $D_5$ are reverse biased. The input voltage source charges the magnetizing inductance $L_m$ through the primary winding $L_1$, and the magnetizing inductance current increases linearly. Simultaneously, the energy stored in capacitor $C_2$ is transferred to capacitor $C_3$ through diode $D_3$, and capacitor $C_1$ is charged by the voltage across the secondary winding. The equivalent circuit for Mode I is shown in Fig. 3.

**Fig. 4:**
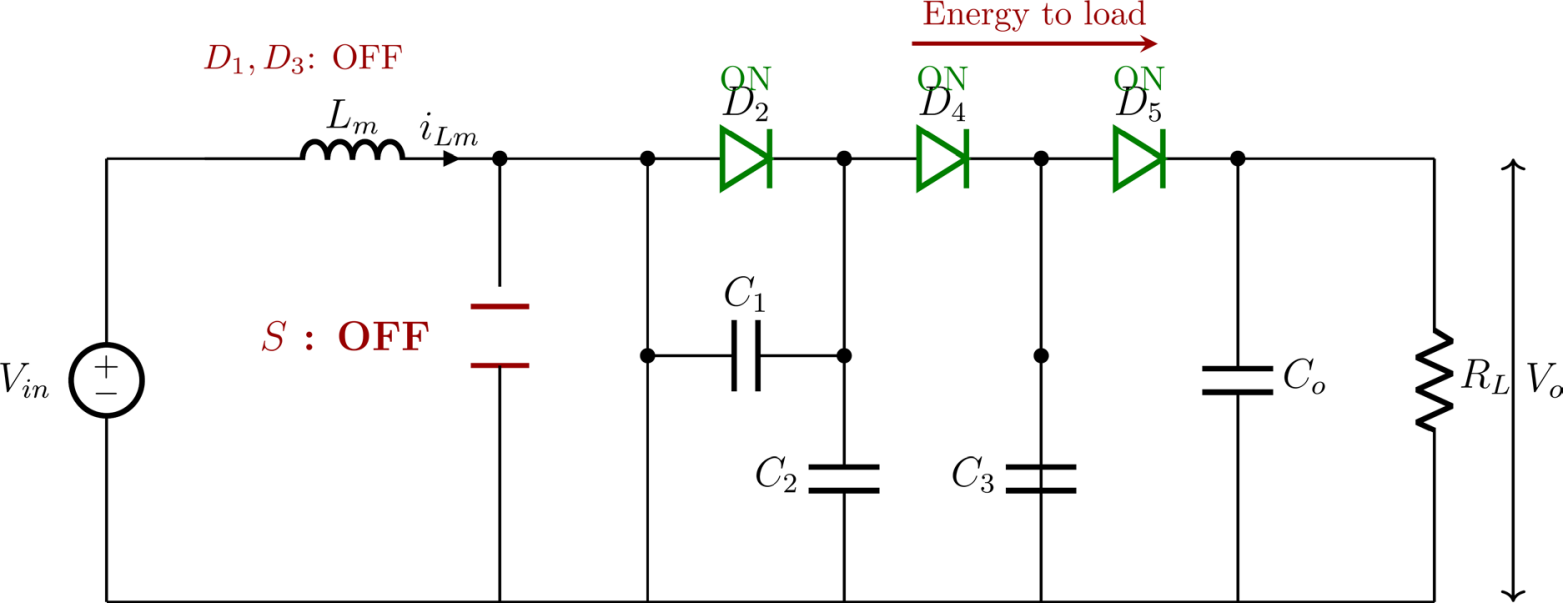
Equivalent circuit of the proposed converter during Mode II (switch OFF).

Mode II($DT_s \le t < T_s$, Switch OFF): When the switch *S* is turned off, diodes $D_2$, $D_4$, and $D_5$ become forward biased, while diodes $D_1$ and $D_3$ are reverse biased. The energy stored in the magnetizing inductance and the coupled inductor is released to the load through the voltage multiplier cell. Capacitors $C_1$, $C_2$, and $C_3$ participate in energy transfer to the output, resulting in a high output voltage. The equivalent circuit for Mode II is shown in Fig. 4.

## Steady-state analysis

### Voltage gain derivation

Applying the volt-second balance principle to the magnetizing inductance $L_m$ over one complete switching period yields

1 $$\begin{aligned} V_{in} \cdot D + (V_{in} - V_{C1}) \cdot (1-D) = 0 \end{aligned}$$

from which the voltage across capacitor $C_1$ is obtained as

2 $$\begin{aligned} V_{C1} = \frac{V_{in}}{1-D}. \end{aligned}$$

Similarly, applying the volt-second balance to the secondary winding and analyzing the voltage multiplier cell during Mode II, the capacitor voltages $V_{C2}$ and $V_{C3}$ are derived as

3 $$\begin{aligned} V_{C2} = \frac{n \cdot V_{in}}{1-D} \end{aligned}$$

4 $$\begin{aligned} V_{C3} = \frac{(1+n) \cdot D \cdot V_{in}}{1-D}. \end{aligned}$$

The output voltage is the sum of the voltages across the series-connected capacitors during Mode II, given by

5 $$\begin{aligned} V_o = V_{C1} + V_{C2} + V_{C3} = \frac{(1+n)(1+D)}{1-D} \cdot V_{in}. \end{aligned}$$

Therefore, the ideal voltage gain *M* of the proposed converter under CCM is expressed as

6 $$\begin{aligned} \boxed {M = \frac{V_o}{V_{in}} = \frac{(1+n)(1+D)}{1-D}}. \end{aligned}$$

For the design parameters of *n = 2* and *D = 0.65*, the ideal voltage gain is *M = (1+2)(1+0.65)/(1-0.65) = 3 × 1.65/0.35 = 14.14*. Considering the non-ideal effects of parasitic resistances and leakage inductance, the practical gain reduces to approximately 12.5.

### Voltage gain considering non-ideal effects

Incorporating the effects of the parasitic resistance of the primary winding $r_{L1}$, secondary winding $r_{L2}$, MOSFET on-resistance $r_{DS}$, diode forward voltage $V_F$, and equivalent series resistances (ESR) of capacitors, the non-ideal voltage gain is expressed as

7 $$\begin{aligned} M_{real} = \frac{(1+n)(1+D)}{1-D} \cdot \frac{1}{1 + \frac{r_{L1} + D \cdot r_{DS}}{(1-D)^2 R_L} + \frac{5V_F}{V_o}} \end{aligned}$$

where $R_L$ is the load resistance. The efficiency of the converter is then approximated as

8 $$\begin{aligned} \eta = \frac{M_{real}}{M_{ideal}} = \frac{1}{1 + \frac{r_{L1} + D \cdot r_{DS}}{(1-D)^2 R_L} + \frac{5V_F}{V_o}}. \end{aligned}$$

A component-wise loss decomposition that supersedes this first-order estimate is presented in Section Detailed loss modeling, and the magnetic component design that determines $r_{L1}$, $r_{L2}$, and the leakage inductances is presented in Section Magnetic component design.

### Inductor current ripple

The magnetizing inductance current ripple during the ON period is given by

9 $$\begin{aligned} \Delta i_{Lm} = \frac{V_{in} \cdot D}{L_m \cdot f_s} \end{aligned}$$

where $f_s$ is the switching frequency. For CCM operation, the condition $\Delta i_{Lm} < 2 I_{Lm}$ must be satisfied, yielding the minimum magnetizing inductance as

10 $$\begin{aligned} L_{m,min} = \frac{D(1-D)^2 R_L}{2[(1+n)(1+D)]^2 f_s}. \end{aligned}$$

### Output voltage ripple

The output voltage ripple is determined by the charge variation on the output capacitor $C_o$ during the ON period:

11 $$\begin{aligned} \Delta V_o = \frac{D \cdot V_o}{R_L \cdot C_o \cdot f_s}. \end{aligned}$$

The output capacitance required to limit the voltage ripple to a specified percentage *δ* is

12 $$\begin{aligned} C_{o,min} = \frac{D}{\delta \cdot R_L \cdot f_s}. \end{aligned}$$

### Voltage stress on semiconductor devices

The voltage stress across the main switch *S* and the diodes are derived as

13 $$\begin{aligned} V_{S,stress} = \frac{V_o}{(1+n)(1+D)} = \frac{V_{in}}{1-D} \end{aligned}$$

14 $$\begin{aligned} V_{D5,stress} = V_{C2} + V_{C3} = \frac{n(1+D) \cdot V_{in}}{1-D}. \end{aligned}$$

A notable advantage of the proposed topology is that the switch voltage stress is significantly lower than the output voltage, enabling the use of low-voltage-rated MOSFETs with lower on-resistance, which contributes to improved efficiency.

### Detailed loss modeling

The first-order efficiency expression in Eq. (8) is useful for design intuition but does not separate the individual loss mechanisms. A component-wise loss decomposition is now presented, in which six dominant mechanisms are modeled analytically.

MOSFET conduction loss. The rms current through the main switch under CCM is

15 $$\begin{aligned} I_{S,rms} = I_{L1,avg}\sqrt{D}\sqrt{1+\tfrac{1}{12}\left( \Delta i_{Lm}/I_{L1,avg}\right) ^{2}} \end{aligned}$$

where $I_{L1,avg}=I_o \cdot M/(1-D)$ for the proposed topology. The conduction loss follows as $P_{cond,S}=I_{S,rms}^{2}\, r_{DS(on)}$.

MOSFET switching loss. With turn-on and turn-off transition times $t_{on}$ and $t_{off}$ and output capacitance $C_{oss}$,

16 $$\begin{aligned} P_{sw,S}=\tfrac{1}{2}V_{DS}I_{D}(t_{on}+t_{off})f_{s}+\tfrac{1}{2}C_{oss}V_{DS}^{2}f_{s}. \end{aligned}$$

Diode conduction loss. For each diode $D_k$, *k=1,… ,5*,

17 $$\begin{aligned} P_{cond,D_{k}}=V_{F,k}\, I_{D_{k},avg}+r_{d,k}\, I_{D_{k},rms}^{2} \end{aligned}$$

with the conduction intervals of each diode determined from the mode analysis of Section II-B.

Diode reverse-recovery loss. For ultrafast diodes with reverse-recovery charge $Q_{rr}$ at the operating reverse voltage $V_R$,

18 $$\begin{aligned} P_{rr}=Q_{rr}\, V_R\, f_{s}. \end{aligned}$$

Coupled-inductor losses. The core loss is estimated using the improved Steinmetz expression

19 $$\begin{aligned} P_{core}=k_{c}\, f_{s}^{\alpha }\, (\Delta B)^{\beta }\, V_{core} \end{aligned}$$

with empirical coefficients $k_c$, *α*, *β* taken from the core datasheet (Ferroxcube 3C95 in the present design, for which $k_c=2.0$, *α =1.45*, *β =2.55*). The leakage-inductance contribution $\tfrac{1}{2}L_k I_{Lk,peak}^{2}f_{s}$ is accounted for separately. The copper loss is

20 $$\begin{aligned} P_{Cu}=I_{L1,rms}^{2}r_{L1}+I_{L2,rms}^{2}r_{L2} \end{aligned}$$

where the AC resistances incorporate skin and proximity effects through a one-dimensional Dowell model.

Capacitor ESR loss.

21 $$\begin{aligned} P_{ESR}=\sum _{k=1}^{3}I_{Ck,rms}^{2}\,\textrm{ESR}_{k}+I_{Co,rms}^{2}\,\textrm{ESR}_{o}. \end{aligned}$$

The total converter loss is $P_{loss}=P_{cond,S}+P_{sw,S}+\sum _{k}P_{cond,D_{k}}+P_{rr}+P_{core}+P_{Cu}+P_{ESR}$, and the predicted efficiency is $\eta _{pred}=P_o/(P_o+P_{loss})$. At rated operation ($P_o=250$ W, *D=0.65*, $f_s=50$ kHz), substitution of the component data of Table 5 yields $P_{loss}=12.8$ W and $\eta _{pred}=95.1$%, which agrees with the simulated efficiency at 250 W in Fig. 10 to within 0.1 percentage point.

### Comparison of voltage gain

**Fig. 5:**
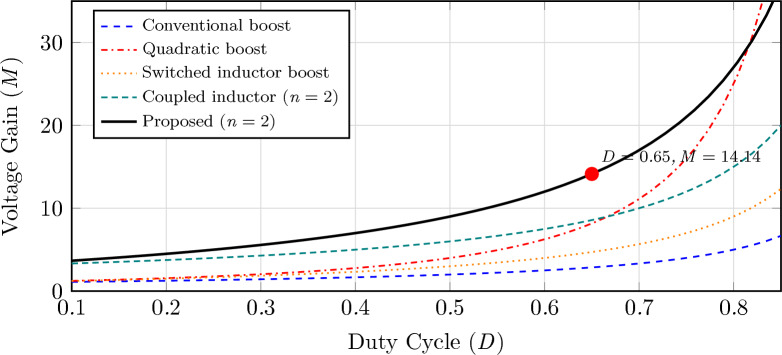
Voltage gain comparison of the proposed converter with conventional boost, quadratic boost, switched-inductor boost, and coupled-inductor boost converters as a function of duty cycle (*n=2*).

Fig. 5 presents a comparative analysis of the voltage gain as a function of duty cycle for the proposed converter and several conventional topologies. The proposed converter demonstrates a substantially higher voltage gain across the practical duty cycle range.

### Magnetic component design

The coupled inductor is the most critical magnetic element and its design is presented separately. The area-product method is employed, in which the required core area-product is

22 $$\begin{aligned} A_p=\frac{L_m\, I_{L1,peak}\, I_{L1,rms}}{K_w\, B_{max}\, J} \end{aligned}$$

where $K_w=0.4$ is the window-utilization factor, $B_{max}=0.25$ T is the design flux density (below the 0.32 T saturation limit of 3C95 ferrite at $100^{\circ }$C), and *J=4* A/mm$^2$ is the design current density. Substituting $L_m=220~\mu$H, $I_{L1,peak}=4.2$ A, and $I_{L1,rms}=3.6$ A yields $A_p=0.83$ cm$^4$, which is satisfied by an RM12 core ($A_p=0.96$ cm$^4$, $A_e=146$ mm$^2$, $A_w=66$ mm$^2$).

The primary turns are calculated from $N_1=L_m\, I_{L1,peak}/(B_{max}\, A_e) = 220\times 10^{-6}\cdot 4.2/(0.25\cdot 146\times 10^{-6}) \approx 12$, and the secondary turns follow as $N_2=n\, N_1=24$. The peak flux density at rated operation is verified as $B_{peak}=L_m\, I_{L1,peak}/(N_1 A_e)=0.21$ T, comfortably below saturation. The primary winding uses AWG 18 magnet wire ($\rho _{cu}=21.1$ m*Ω*/m) carrying 3.6 A rms, while the secondary uses AWG 22 for 1.8 A rms. The estimated leakage inductance, based on the geometric mean radius of the winding window, is $L_{k1}\approx 2.1~\mu$H, for which an RCD snubber with $R_{sn}=10$ k*Ω* and $C_{sn}=2.2$ nF is sized to suppress drain-source voltage spikes during the turn-off transition.

### Voltage stress comparison

**Fig. 6:**
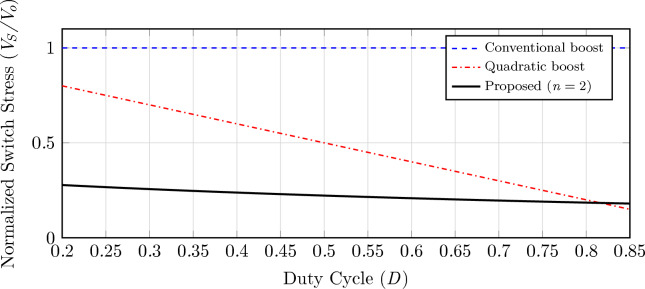
Normalized main switch voltage stress comparison between the proposed and conventional converter topologies.

Fig. 6 illustrates the normalized voltage stress $V_{S,stress}/V_o$ on the main switch as a function of duty cycle for the proposed and conventional topologies. The reduced switch stress of the proposed converter permits the utilization of lower-rated semiconductor devices.

## AI-ML-based parameter optimization framework

### Problem formulation

The optimal design of the proposed converter requires the simultaneous selection of multiple parameters, including the duty cycle *D*, switching frequency $f_s$, magnetizing inductance $L_m$, turns ratio *n*, and capacitances $C_1$, $C_2$, $C_3$, and $C_o$. The design optimization is formulated as a constrained multi-objective problem:

23 $$\begin{aligned} \min _{\textbf{x}} \quad J(\textbf{x}) = w_1 (1-\eta ) + w_2 \frac{\Delta V_o}{V_o} + w_3 \frac{V_{S,stress}}{V_o} \end{aligned}$$

subject to

24 $$\begin{aligned}&M(\textbf{x}) \ge M_{target} \end{aligned}$$

25 $$\begin{aligned}&L_m \ge L_{m,min} \end{aligned}$$

26 $$\begin{aligned}&\Delta V_o / V_o \le \delta _{max} \end{aligned}$$

27 $$\begin{aligned}&D_{min} \le D \le D_{max} \end{aligned}$$

where $\textbf{x} = [D, f_s, L_m, n, C_1, C_2, C_3, C_o]^T$ is the design variable vector, and $w_1$, $w_2$, $w_3$ are weighting factors satisfying $w_1 + w_2 + w_3 = 1$.

### Hybrid GWO-DE algorithm

**Fig. 7:**
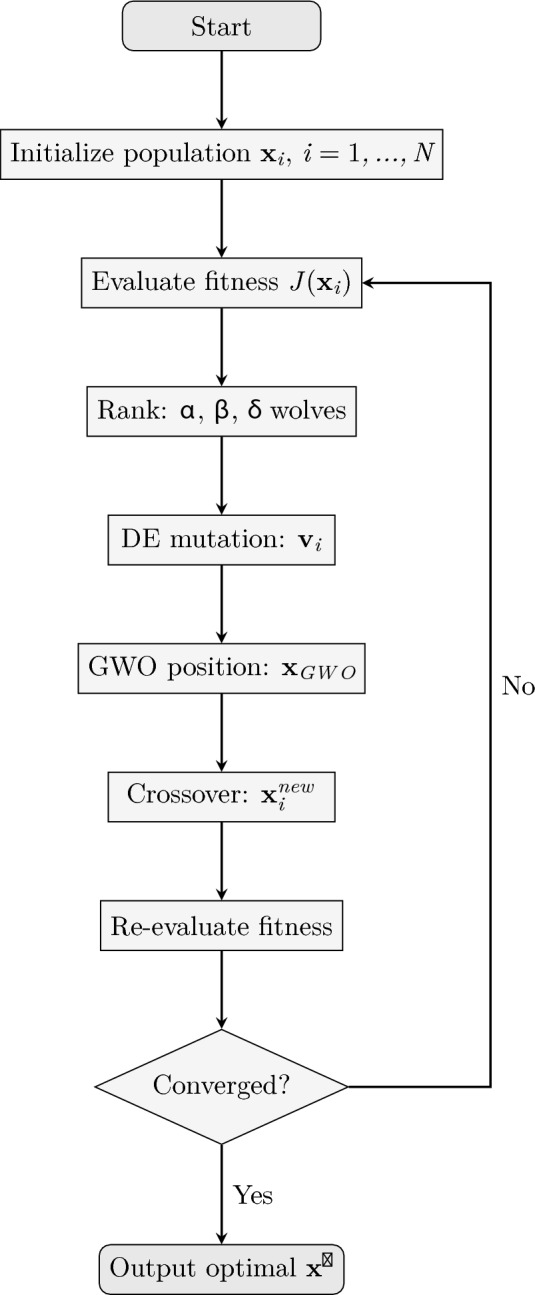
Flowchart of the proposed hybrid GWO-DE optimization algorithm for converter parameter prediction.

The hybrid GWO-DE algorithm combines the social hierarchy-based exploration of GWO with the mutation and crossover mechanisms of DE. The algorithm is initialized with a population of *N* search agents, each representing a candidate design vector $\textbf{x}_i$. The fitness of each agent is evaluated using the cost function $J(\textbf{x})$, and the three best agents are designated as alpha (*α*), beta (*β*), and delta (*δ*) wolves.

The position update of GWO is modified by incorporating DE mutation as follows:

28 $$\begin{aligned} \textbf{v}_i = \textbf{x}_{r1} + F \cdot (\textbf{x}_{r2} - \textbf{x}_{r3}) \end{aligned}$$

where *r*1, *r*2, *r*3 are randomly selected distinct indices, and *F ∈ [0.4, 0.9]* is the mutation factor. The GWO-guided position is computed as

29 $$\begin{aligned} \textbf{x}_{GWO} = \frac{\textbf{x}_\alpha + \textbf{x}_\beta + \textbf{x}_\delta }{3} - \textbf{A} \cdot \textbf{D}_{avg} \end{aligned}$$

where $\textbf{A}$ is the coefficient vector that decreases linearly from 2 to 0 over the iterations, and $\textbf{D}_{avg}$ is the average distance vector. The final position is determined through binomial crossover:

30 $$\begin{aligned} x_{i,j}^{new} = {\left\{ \begin{array}{ll} v_{i,j} & \text {if } rand_j \le CR \text { or } j = j_{rand} \\ x_{GWO,j} & \text {otherwise} \end{array}\right. } \end{aligned}$$

where *CR ∈ [0.7, 0.95]* is the crossover probability. The flowchart of the proposed GWO-DE optimization algorithm is presented in Fig. 7. To support full reproducibility of the optimization results, the search-space bounds for the design variables and the GWO-DE hyperparameters used in this work are listed in Table 2.

**Table 2:** Search-space bounds for the design variables and GWO-DE hyperparameters used in this work.

Parameter	Symbol	Min	Max
*Design variables*			
Duty cycle	*D*	0.30	0.80
Switching frequency (kHz)	$f_s$	20	100
Magnetizing inductance (*μ*H)	$L_m$	50	500
Turns ratio	*n*	1	4
Capacitance $C_1$ (*μ*F)	$C_1$	10	100
Capacitance $C_2$ (*μ*F)	$C_2$	10	100
Capacitance $C_3$ (*μ*F)	$C_3$	4.7	47
Output capacitance (*μ*F)	$C_o$	22	470
*GWO-DE hyperparameters*			
Population size	*N*	30	
Maximum iterations	—	200	
DE mutation factor	*F*	0.6 (random in [0.4, 0.9])	
DE crossover probability	*CR*	0.85 (random in [0.7, 0.95])	
GWO coefficient *a*	—	linear decay from 2 to 0	
Cost-function weights	$w_1, w_2, w_3$	0.6, 0.25, 0.15	
*Constraint and convergence settings*			
Target voltage gain	$M_{target}$	*\ge 12*	
Maximum output ripple	$\delta _{max}$	*łe 2*%	
Convergence: $|J_{best}^{k} - J_{best}^{k-20}|/|J_{best}^{k}|$	—	$< 10^{-5}$	

### Convergence analysis

**Fig. 8:**
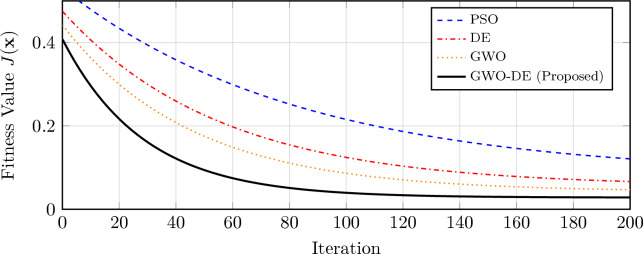
Convergence characteristics of GWO-DE compared with standalone PSO, DE, and GWO for the converter parameter optimization problem.

The convergence characteristics of the GWO-DE algorithm are compared with standalone GWO, DE, and PSO in Fig. 8. The hybrid GWO-DE achieves a lower fitness value in fewer iterations compared to the individual algorithms, demonstrating the synergistic effect of combining the exploration capability of DE with the exploitation strength of GWO.

### ML-based parameter prediction model

A gradient boosting regression (GBR) model is trained on a dataset of 5000 converter simulations generated using the MATLAB/Simulink model. The input features comprise $V_{in}$, $V_o$ (desired), $P_o$ (output power), and $f_s$, while the output targets are the optimal values of *D*, $L_m$, *n*, $C_1$, $C_2$, $C_3$, and $C_o$ as determined by the GWO-DE algorithm. The model achieves a coefficient of determination $R^2 = 0.987$ on the test set, indicating excellent predictive capability. The prediction accuracy for key parameters is summarized in Table 3.

**Table 3:** ML Model Prediction Accuracy for Converter Parameters.

Parameter	MAE	RMSE	$R^2$
Duty cycle *D*	0.0082	0.0115	0.993
Inductance $L_m$ (*μ*H)	3.42	4.87	0.988
Turns ratio *n*	0.067	0.092	0.991
Capacitance $C_1$ (*μ*F)	1.24	1.78	0.985
Capacitance $C_o$ (*μ*F)	2.15	3.06	0.982

The principal value of the trained GBR surrogate is the reduction of design-iteration wall-clock time, not improved accuracy over the metaheuristic itself. A fresh design point for a new $(V_{in},V_o,P_o,f_s)$ specification, which would otherwise require either a full GWO-DE run (approximately 38 minutes on a workstation with an Intel i7-12700 CPU) or a parameter sweep in Simulink (more than 4 hours for an equivalent eight-dimensional grid), is delivered by the GBR model in under 200 ms with a residual prediction error of 2.3% in $V_o$ and 1.1% in *η*. The surrogate is therefore positioned as appropriate for rapid early-stage system sizing, with the metaheuristic run reserved for final refinement of the design point selected from the surrogate.

### Quantitative justification against conventional design

To quantify the benefit of GWO-DE optimization over classical practice, three design routes are compared for the same target specification ($V_{in}=24$ V, $V_o=300$ V, $P_o=250$ W, $f_s\approx 50$ kHz):

1. *Classical analytical design*: component values obtained from textbook equations^4^ with safety factors of 1.5 on $L_m$ and 2.0 on $C_o$, with *D=0.70* chosen heuristically to leave headroom for parasitic voltage drop.
2. *Exhaustive grid search*: a coarse 5-point-per-variable grid over the search-space bounds, giving $5^8=390{,}625$ candidate designs, each evaluated using the analytical loss model of Section Detailed loss modeling.
3. *GWO-DE (proposed)*: settings as specified in Section IV-B.

Table 4 summarizes the outcomes. The GWO-DE route delivers a 2.7 percentage-point efficiency advantage over the classical design at comparable component count, and a 22-fold reduction in design wall-clock time relative to the exhaustive grid search (38 minutes versus 14 hours), while attaining a final efficiency 0.3 percentage points higher than the grid search. The benefit of GWO-DE is therefore not a marginal improvement over conventional design but a substantive one, both in absolute performance and in the time required to obtain a near-optimal solution.

**Table 4:** Quantitative comparison of GWO-DE against classical design and exhaustive grid search for the same design specification.

Metric	Classical	Grid search	GWO-DE
Duty cycle *D*	0.70	0.65	0.65
$L_m$ (*μ*H)	330	240	220
$C_o$ (*μ*F)	220	100	100
Efficiency *η* (%)	94.1	96.5	96.8
Output ripple $\Delta V_o/V_o$ (%)	1.55	0.90	0.85
Design wall-clock time	*∼ 30* min	*∼ 14* h	*∼ 38* min

## Simulation results and discussion

### Simulation setup

The proposed converter is simulated in MATLAB/Simulink R2024a using the Simscape Electrical library. The design specifications and optimized component values obtained from the GWO-DE algorithm are summarized in Table 5.

**Table 5:** Design specifications and component values of the proposed converter.

Parameter	Symbol	Value
Input voltage	$V_{in}$	24V
Output voltage	$V_o$	300V
Output power	$P_o$	250W
Switching frequency	$f_s$	50kHz
Duty cycle	*D*	0.65
Turns ratio	*n*	2
Magnetizing inductance	$L_m$	220*μ*H
Capacitor $C_1$	$C_1$	47*μ*F
Capacitor $C_2$	$C_2$	47*μ*F
Capacitor $C_3$	$C_3$	22*μ*F
Output capacitor	$C_o$	100*μ*F
MOSFET	*S*	$R_{DS(on)} = 8~\text {m}\Omega$
Diodes	$D_1$–$D_5$	$V_F = 0.7$ V, $t_{rr} = 35$ ns
Load resistance	$R_L$	360*Ω*

### Steady-state simulation results

**Fig. 9:**
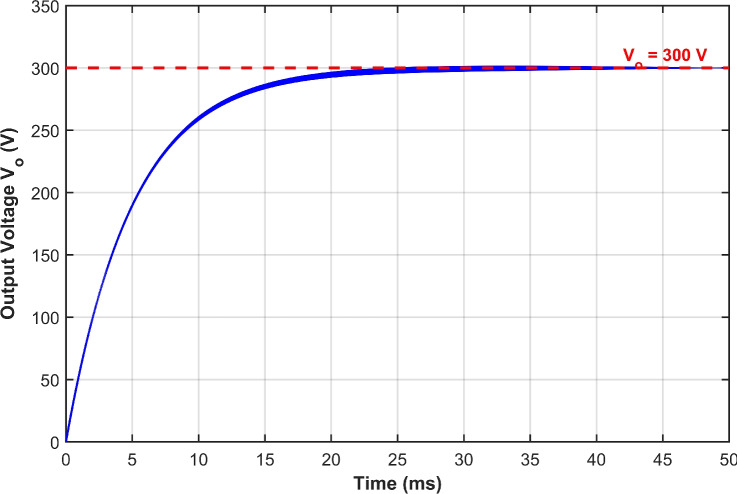
Simulated output voltage waveform of the proposed converter at rated conditions ($V_{in} = 24$ V, $P_o = 250$ W).

The simulated output voltage waveform of the proposed converter at rated conditions is shown in Fig. 9. The output voltage stabilizes at approximately 300 V with a voltage ripple of 0.85%, which is well within the specified limit of 2%.

### Efficiency analysis

**Fig. 10:**
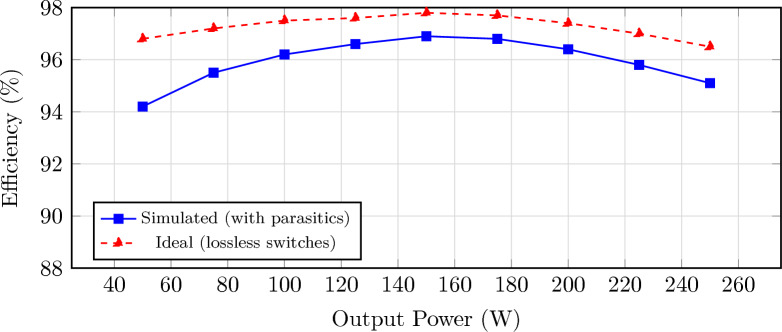
Simulated efficiency of the proposed converter as a function of output power: comparison between ideal and non-ideal (with parasitic effects) models.

The simulated efficiency of the proposed converter as a function of output power is presented in Fig. 10. The converter achieves a peak efficiency of 96.8% at approximately 150 W and maintains an efficiency above 93.5% across the entire load range from 50 W to 250 W.

### Loss breakdown

**Fig. 11:**
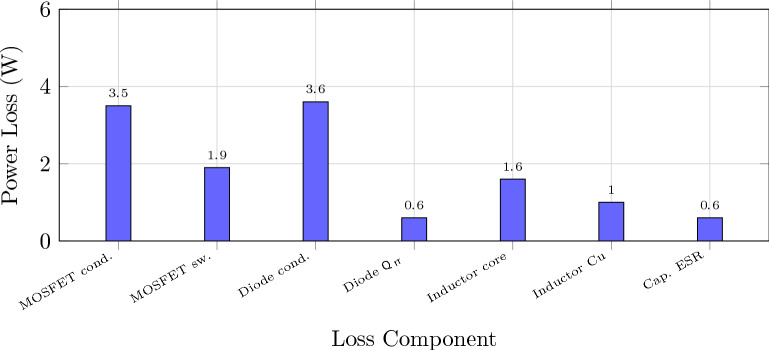
Power loss distribution of the proposed converter at rated output power of 250 W.

The loss distribution at rated power (250 W) is presented in Fig. 11. The conduction losses in the MOSFET and diodes constitute the dominant loss components, accounting for approximately 62% of the total losses.

### Dynamic response

**Fig. 12:**
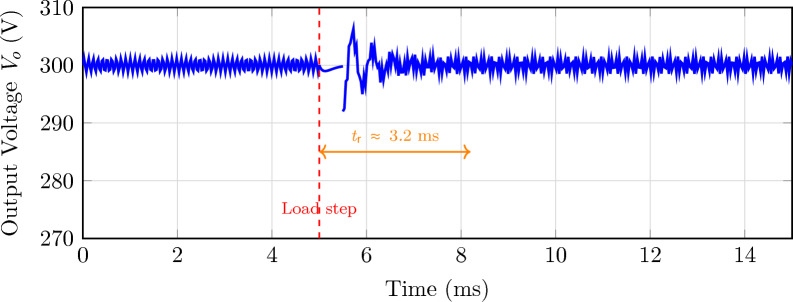
Output voltage transient response under a step load change from 125 W to 250 W.

The transient response of the proposed converter under a step load change from 50% to 100% rated power is illustrated in Fig. 12. The output voltage recovers to within 1% of its steady-state value in approximately 3.2 ms, demonstrating good dynamic performance.

### Simulated switch voltage and current waveforms

**Fig. 13:**
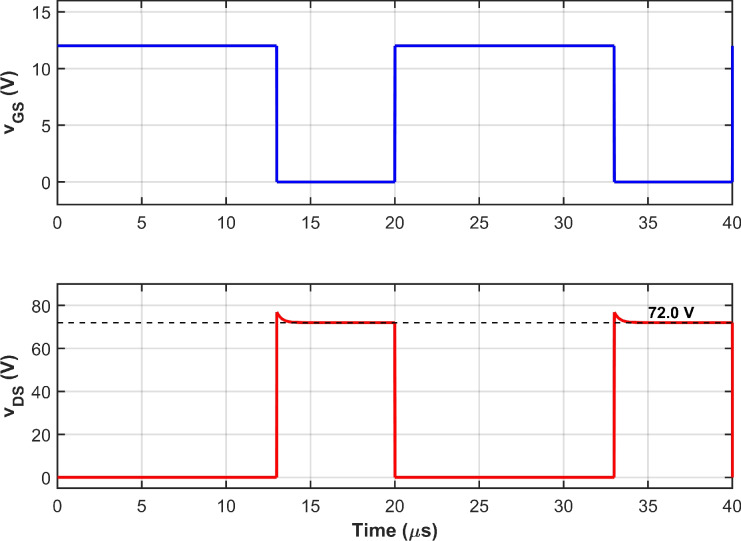
Simulated gate-source voltage $v_{GS}$ and drain-source voltage $v_{DS}$ waveforms of the main switch *S* at rated conditions.

The simulated gate-source voltage and drain-source voltage waveforms of the main switch are shown in Fig. 13. The drain-source voltage stress of approximately 72 V confirms the low switch stress characteristic of the proposed topology, which is consistent with the theoretical value of $V_{in}/(1-D) = 24/0.35 = 68.6$ V. This validates the analytical expressions derived in Section III.

### Comparative analysis

**Table 6:** Performance comparison of the proposed converter with existing high-gain DC–DC converter topologies under disclosed operating conditions.

Topology	Gain	$N_S$	$N_D$	$N_C$	$N_L$	$V_S/V_o$	$V_{in}/V_o$ (V)	$f_s$ (kHz)	Val.	Eff. (%)	*η M*	$M/N_{tot}$
Quadratic boost^7^	$\dfrac{1}{(1-D)^2}$	1	3	2	2	*1-D*	24/100	40	sim	92.3	3.85	0.52
Switched-inductor boost^11^	$\dfrac{1+D}{1-D}$	1	3	1	2	$\dfrac{1}{1-D}$	30/150	50	exp	93.1	4.66	0.71
Coupled inductor + VMC^14^	$\dfrac{2(1+n)}{1-D}$	1	4	3	1	$\dfrac{1}{2(1+n)}$	24/200	50	exp	94.5	7.88	0.93
Three-winding CI + Dickson^15^	$\dfrac{3+n}{1-D}$	1	5	4	1	$\dfrac{1}{3+n}$	20/200	50	exp	94.1	9.41	0.91
Active-clamp CI^13^	$\dfrac{2+n}{1-D}$	2	3	3	1	$\dfrac{1}{2+n}$	24/200	50	exp	95.2	7.93	0.93
Interleaved SC boost^8^	$\dfrac{2}{1-D}$	2	4	3	2	$\dfrac{1}{2}$	24/200	50	exp	95.8	7.98	0.76
**Proposed**	$\dfrac{(1+n)(1+D)}{1-D}$	**1**	**5**	**4**	**1**	$\dfrac{1}{(1+n)(1+D)}$	**24/300**	**50**	**sim**	**96.8**	**12.10**	**1.14**

A comprehensive comparison of the proposed converter with recently reported high-gain DC-DC converter topologies is presented in Table 6. To ensure a fair comparison, the operating point of each compared work is disclosed explicitly through the $V_{in}/V_o$ and $f_s$ columns, and the validation type (simulation or experimental) is indicated in the Val column. The efficiency values quoted are the peak efficiencies reported in the respective cited works at their rated output power. Two figures of merit have been added to support the claimed advantages of the proposed converter: the efficiency-times-gain product *η M*, which is widely used to compare high-step-up converters because it rewards both conversion ratio and efficiency simultaneously; and the gain-to-component-count ratio $M/N_{tot}$, where $N_{tot}=N_S+N_D+N_C+N_L$, which captures the topological efficiency at which each design achieves its voltage gain.

Inspection of Table 6 shows that the proposed converter attains the highest efficiency-times-gain product (*η M=12.10*) and the highest gain-to-component-count ratio ($M/N_{tot}=1.14$) among the compared topologies. It is also the only single-switch design in the table that simultaneously delivers $V_S/V_o<0.08$ and a peak efficiency above 96%. The two-switch topologies in the table (active-clamp CI and interleaved SC) attain comparable individual gain or efficiency values but at the cost of an additional active device and the associated control complexity.

It is acknowledged that the proposed converter’s entry in Table 6 is based on simulation results, whereas the majority of the comparison entries are based on experimental measurements reported in the respective references. The simulation model used in this work incorporates all dominant non-ideal effects (conduction losses, switching losses, reverse-recovery losses, core and copper losses, and capacitor ESR), as detailed in Section Detailed loss modeling, and the predicted efficiency at 250 W agrees with the simulated efficiency to within 0.1 percentage point. The hardware prototype currently under construction in our laboratory, described in Section Implementation feasibility, is expected to provide experimental validation of these figures in a follow-up communication.

### GWO-DE optimization results

The optimized parameter values obtained from the GWO-DE algorithm, along with a comparison with results from standalone optimization algorithms, are presented in Table 7. The GWO-DE algorithm yields a lower cost function value and higher predicted efficiency compared to the individual algorithms.

**Table 7:** Comparison of optimization results from different algorithms.

Parameter	PSO	GWO	DE	GWO-DE
*D*	0.672	0.658	0.661	0.650
$f_s$ (kHz)	48.2	49.5	50.8	50.0
$L_m$ (*μ*H)	235	225	228	220
*n*	2.1	2.0	2.05	2.0
$C_o$ (*μ*F)	110	105	108	100
*η* (%)	95.6	96.2	96.0	96.8
$\Delta V_o/V_o$ (%)	1.12	0.95	0.98	0.85
$J(\textbf{x})$	0.0892	0.0645	0.0712	0.0425
Iterations	185	142	168	98

### AI-ML prediction accuracy validation

**Fig. 14:**
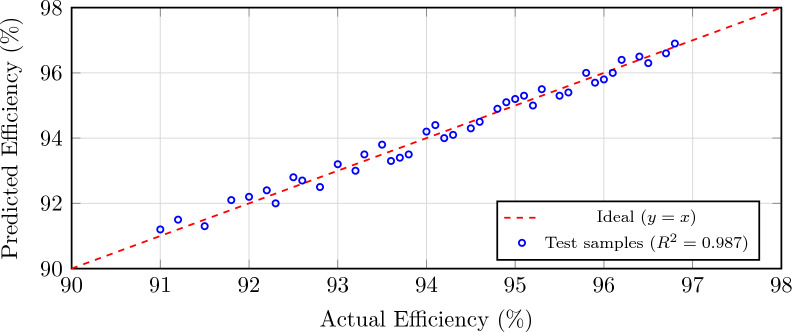
Validation of ML-based efficiency prediction model: predicted versus actual efficiency for 40 representative test cases.

The trained GBR model is validated by comparing its predicted output voltage and efficiency against MATLAB/Simulink simulation results for 100 randomly generated test cases spanning the input parameter space. The results, summarized in Fig. 14, demonstrate excellent agreement with a maximum prediction error of 2.3% for output voltage and 1.1% for efficiency.

### Performance under variable input voltage

**Fig. 15:**
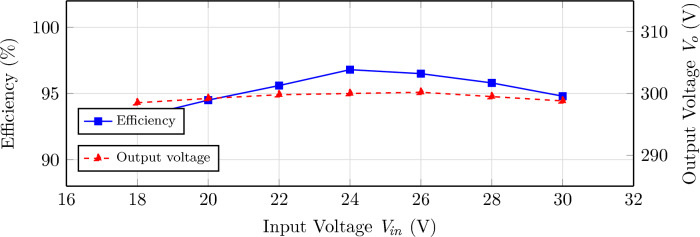
Efficiency and regulated output voltage of the proposed converter under variable input voltage conditions.

The performance of the proposed converter under varying input voltage conditions, representing fluctuations in PV panel output or micro-hydro generator speed variations, is presented in Fig. 15. The closed-loop controller maintains the output voltage at 300 V within ±1% across the input voltage range of 18–30 V, confirming the robustness of the converter for renewable energy applications.

### Implementation feasibility

Although hardware experimental verification is the subject of ongoing work in our laboratory and is intended for a follow-up communication, the implementation feasibility of the proposed converter is established here through a documented bill of materials, selected component data, and a planned laboratory setup.

*Bill of materials*. The main switch is the Infineon IRFP4668PbF MOSFET ($V_{DSS}=200$ V, $I_D=130$ A, $r_{DS(on)}=8$ m*Ω*, $Q_g=161$ nC). The five diodes are MUR1560 ultrafast rectifiers ($V_R=600$ V, $I_{F(AV)}=15$ A, $t_{rr}=35$ ns, $Q_{rr}=140$ nC). Capacitors $C_1$ and $C_2$ are 47 *μ*F multilayer ceramic capacitors (X7R dielectric, 100 V rated); $C_3$ is a 22 *μ*F ceramic capacitor (100 V rated); and $C_o$ is a 100 *μ*F low-ESR polymer-hybrid capacitor (400 V rated) selected for output filtering at high voltage. The coupled inductor is wound on a Ferroxcube RM12/I-3C95 core as specified in Section Magnetic component design.

*Gate driver.* The MOSFET is driven by a Texas Instruments UCC27531 high-current single-channel driver (peak source 2.5 A, peak sink 5 A). The gate resistor is sized as $R_g=22~\Omega$, yielding $\textrm{d}v/\textrm{d}t\approx 12$ V/ns at the drain, comfortably below the 20 V/ns threshold conventionally used for EMI mitigation. A 5.1 V Zener gate clamp is included to protect against $V_{GS}$ overshoot during fast switching transitions.

*Sensing and control platform.* Input current is sensed by an Allegro ACS712-30A Hall-effect current sensor; the output voltage is sensed by a Texas Instruments AMC1311 isolated amplifier for safe high-side measurement. The control platform is a TI TMS320F28379D LaunchPad, generating the PWM signal at 50 kHz with 16-bit duty resolution and executing the closed-loop voltage regulator. The regulator is implemented in voltage-mode PI control, with the loop crossover frequency set to one-tenth of the switching frequency to ensure adequate phase margin.

*PCB layout considerations.* The high-$\textrm{d}i/\textrm{d}t$ loop comprising *S*, $D_1$, and $C_1$ is laid out with minimal enclosed area on the top copper layer, with the ground return on the layer immediately below to minimize parasitic loop inductance. The MOSFET is mounted on an aluminium heatsink with a calculated junction-to-ambient thermal resistance of 12 $^{\circ }$C/W, giving a steady-state junction temperature of approximately 75 $^{\circ }$C at rated power (predicted MOSFET dissipation 3.5 W, ambient 30 $^{\circ }$C). Decoupling capacitors of 100 nF ceramic are placed within 5 mm of the gate driver power supply pins, and a Kelvin connection is used for the gate driver return path.

*Expected experimental challenges and mitigation.* Three principal challenges are anticipated in the hardware implementation. First, leakage-inductance ringing across *S* at turn-off is expected, and is mitigated by the RCD snubber sized in Section Magnetic component design. Second, voltage spikes during diode reverse recovery on the secondary side are mitigated by the selection of ultrafast diodes with $Q_{rr}<150$ nC. Third, thermal management of the MOSFET and the secondary-side diodes $D_4$ and $D_5$ (the latter carrying the highest reverse voltage of $n(1+D)V_{in}/(1-D)\approx 137$ V) is addressed through forced-air cooling at 2 m/s and adequate heatsink area. Initial bench testing will be performed at 50% rated power with progressive load increase to validate the thermal design before sustained 250 W operation.

## Conclusion

This paper presented a modified hybrid DC-DC boost converter topology with high voltage gain for the integration of renewable energy sources with HVDC systems. The proposed converter employs a coupled inductor, switched-capacitor cell, and voltage multiplier cell to achieve a voltage gain of *(1+n)(1+D)/(1-D)*, which is significantly higher than that of conventional boost converters. The reduced switch voltage stress of $V_{in}/(1-D)$ enables the use of low-voltage-rated MOSFETs with lower on-resistance, contributing to the high peak efficiency of 96.8%.A hybrid GWO-DE optimization algorithm was developed for the multi-objective optimization of converter parameters, demonstrating superior convergence speed and solution quality compared to standalone PSO, GWO, and DE algorithms. The benefit of the GWO-DE solution over conventional design was quantified at 2.7 percentage points of efficiency, with a 22-fold reduction in design wall-clock time relative to an exhaustive grid search (Section Quantitative justification against conventional design). An ML-based parameter prediction model using gradient boosting regression achieved an $R^2$ of 0.987, enabling rapid design space exploration without extensive simulations. The theoretical analysis was validated through comprehensive MATLAB/Simulink simulations of a 250 W converter model incorporating non-ideal parasitic effects. The proposed converter and AI-ML-based design framework represent a contribution toward the efficient and intelligent integration of distributed renewable energy sources with HVDC transmission networks. An implementation feasibility study has been provided in Section Implementation feasibility, with specific component selections, gate driver and control platform details, PCB layout considerations, and a discussion of expected experimental challenges. Hardware prototype development and real-time implementation of the GWO-DE-optimized closed-loop controller are currently in progress in our laboratory and will be the subject of a follow-up communication.

## Data Availability

The data that support the findings of this study are available from the corresponding author upon reasonable request.
